# (Homo)glutathione Deficiency Impairs Root-knot Nematode Development in *Medicago truncatula*

**DOI:** 10.1371/journal.ppat.1002471

**Published:** 2012-01-05

**Authors:** Fabien Baldacci-Cresp, Christine Chang, Mickaël Maucourt, Catherine Deborde, Julie Hopkins, Philippe Lecomte, Stéphane Bernillon, Renaud Brouquisse, Annick Moing, Pierre Abad, Didier Hérouart, Alain Puppo, Bruno Favery, Pierre Frendo

**Affiliations:** 1 Interactions Biotiques et Santé Végétale UMR INRA 1301 -CNRS 6243-Université de Nice-Sophia Antipolis, Sophia Antipolis, France; 2 Université de Bordeaux, UMR 1332 Biologie du Fruit et Pathologie, Centre INRA de Bordeaux, Villenave d'Ornon, France; 3 Metabolome-Fluxome Facility of Bordeaux Functional Genomics Center, IBVM, Centre INRA de Bordeaux, Villenave d'Ornon, France; 4 INRA - UMR 1332 Biologie du Fruit et Pathologie, Centre INRA de Bordeaux, Villenave d'Ornon, France; Virginia Polytechnic Institute and State University, United States of America

## Abstract

Root-knot nematodes (RKN) are obligatory plant parasitic worms that establish and maintain an intimate relationship with their host plants. During a compatible interaction, RKN induce the redifferentiation of root cells into multinucleate and hypertrophied giant cells essential for nematode growth and reproduction. These metabolically active feeding cells constitute the exclusive source of nutrients for the nematode. Detailed analysis of glutathione (GSH) and homoglutathione (hGSH) metabolism demonstrated the importance of these compounds for the success of nematode infection in *Medicago truncatula*. We reported quantification of GSH and hGSH and gene expression analysis showing that (h)GSH metabolism in neoformed gall organs differs from that in uninfected roots. Depletion of (h)GSH content impaired nematode egg mass formation and modified the sex ratio. In addition, gene expression and metabolomic analyses showed a substantial modification of starch and γ-aminobutyrate metabolism and of malate and glucose content in (h)GSH-depleted galls. Interestingly, these modifications did not occur in (h)GSH-depleted roots. These various results suggest that (h)GSH have a key role in the regulation of giant cell metabolism. The discovery of these specific plant regulatory elements could lead to the development of new pest management strategies against nematodes.

## Author Summary

Parasitic nematodes are microscopic worms that cause major diseases of plants, animals and humans. During compatible interactions, root-knot nematodes (RKN) induce the formation of galls in which redifferentiation of root cells into multinucleate and hypertrophied giant cells is essential for nematode growth and reproduction. The importance of glutathione (GSH), a major antioxidant molecule involved in plant development, in plant microbe interaction and in abiotic stress response, was analyzed during the plant-RKN interaction. Our analyses demonstrated that the gall development and functioning are characterized by an adapted GSH metabolism and that depletion of GSH content impairs nematode reproduction and modified sex ratio. This phenotype is linked to specific modifications of carbon metabolism which do not occur in uninfected roots indicating a peculiar metabolism of this neoformed organ. This first metabolomic analysis during the plant-RKN interaction highlights the regulatory role played by GSH in this pathogenic interaction and completes our vision of the role of GSH during plant-pathogen interactions. RKN sex ratio modification has previously been observed under unfavorable nematode feeding conditions suggesting that the GSH-redox system could be a general sensor of gall fitness in natural conditions.

## Introduction

Glutathione (GSH) is a tripeptide, γ-glutamyl-cysteinyl-glycine, present in a wide range of organisms. It is a low molecular weight thiol which in plants is involved in antioxidant defense, detoxification of xenobiotics and tolerance to abiotic and biotic stresses [1]. GSH regulates the expression of stress defense genes and is involved in plant resistance to oomycete and bacterial pathogens and insect herbivores [2]–[4]. GSH is also involved in organ development and its role in seed maturation and root and leaf growth has been established [5]–[7]. In certain legumes, a GSH homolog, homoglutathione (hGSH), is also present instead of, or in addition to, GSH [8]–[10]. The synthesis of GSH is a two-step process. In the first step, γ-glutamylcysteine synthetase (γ-ECS) produces the dipeptide γ-glutamylcysteine (γ-EC) from L-glutamic acid and L-cysteine and regulates the accumulation of GSH and hGSH [(h)GSH]. The formation of GSH and hGSH is determined by the substrate specificity of the enzyme catalyzing the second step. Glutathione synthetase (GSHS) catalyses the addition of glycine to γ-EC, whereas homoglutathione synthetase (hGSHS) catalyses the addition of β-alanine to γ-EC.

In the model legume *Medicago truncatula*, we have shown that (h)GSH deficiency alters the nitrogen fixing symbiotic interaction and reduces the formation of root nodules [11]. The transcriptomic response of (h)GSH-deficient plants to *Sinorhizobium meliloti* infection showed a downregulation of genes involved in meristem formation and an increased expression of several genes involved in the early plant defense reaction against abiotic or biotic stresses [12]. Thus (h)GSH may regulate both nodule neoformation and the plant defense response during symbiosis [12].

Plant-parasitic nematodes that infect *M. truncatula* and other legumes have emerged as models for studying the molecular dialogue during plant-nematode interactions and investigating whether beneficial plant symbionts and biotrophic pathogens induce distinct or overlapping regulatory pathways [13]–[17]. Root-knot nematodes (RKN, *Meloidogyne* spp.) are obligate root pathogens that interact with their hosts in a remarkable manner. During a compatible interaction, infective second stage RKN juvenile (J2) migrate intercellularly towards the vascular cylinder and induce the redifferentiation of root cells into specialized nematode feeding cells named giant cells (GCs). GCs are hypertrophied and multinucleate. They are the result of successive nuclear division without cell division and isotropic growth [18]. Mature GCs are metabolically very active, and act as transfer cells between vascular tissues and RKN. They are the sole source of nutrients for the feeding nematode and are thus essential for nematode growth and development [19]. Hyperplasia of neighboring cells (NCs) leads to the gall, the characteristic symptom of RKN infection. Once sedentarized, J2 molt three times to reach the adult stage. The reproduction of *M. incognita* is parthenogenetic: males migrate from the root and are not required for reproduction whereas the pear-shaped females produce and extrude eggs in a gelatinous matrix onto the root surface.

The formation of both nodule and gall requires root cell dedifferentiation and modification of their cell cycle [20], [21]. Moreover, both nematodes and rhizobia seem to actively modulate the host plant defense, so as to allow the compatible interaction [22], [23]. The modifications to the plant defense and organogenesis observed in these plant-microbe interactions led us to analyze (h)GSH metabolism in galls. We studied the involvement of these tripeptides in the *M. incognita* development cycle in *M. truncatula* and tested for modifications of gall metabolism under (h)GSH deficiency.

## Results

### (h)GSH metabolism is modified in nematode-induced root galls

**Figure 1:**
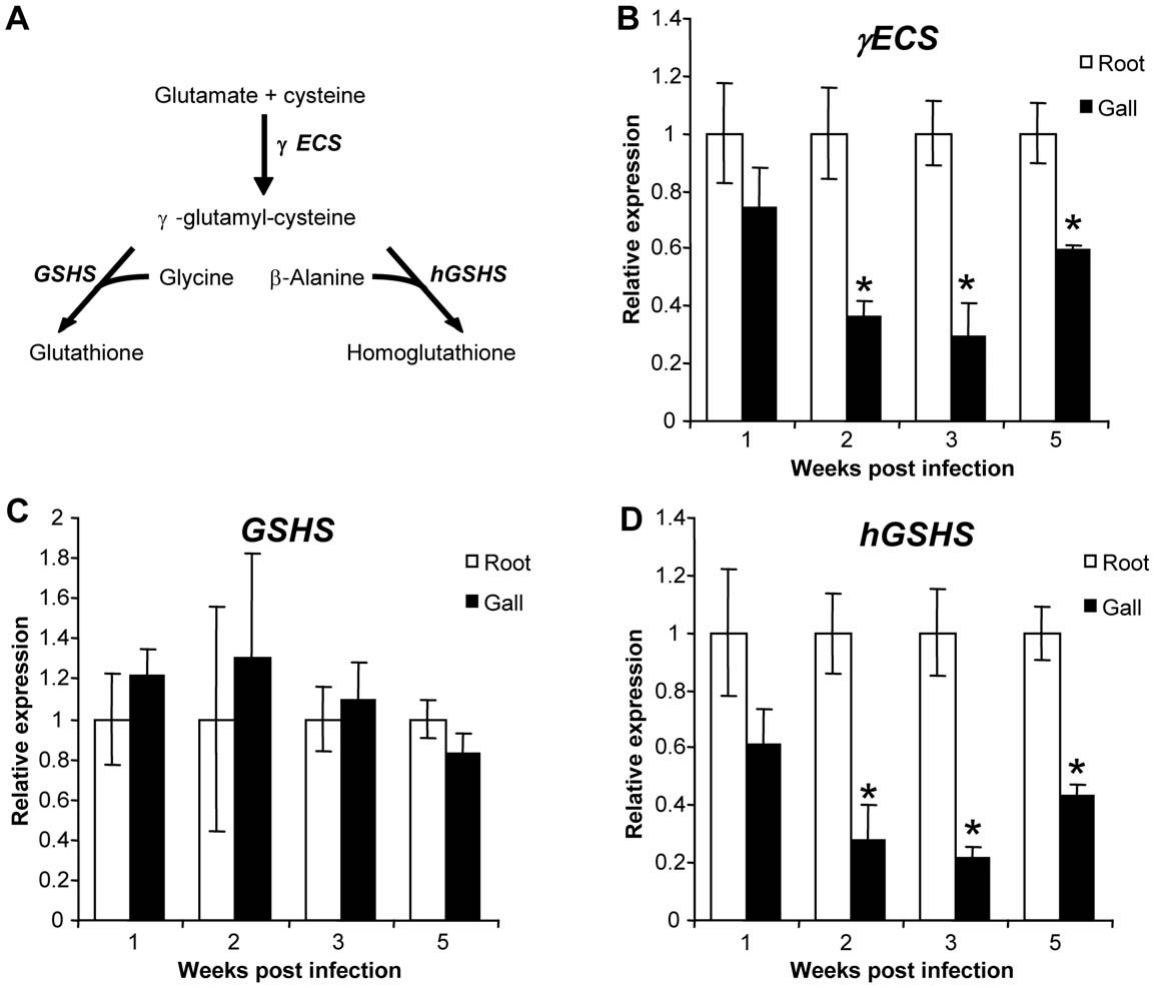
qRT-PCR expression analysis of genes involved in glutathione and homoglutathione synthesis pathway in galls. The synthesis pathway of glutathione and homoglutathione is presented in (A). The expression levels of *M. truncatula* γ*-glutamylcysteine synthetase* (γ*ECS*) (B), *glutathione synthetase* (*GSHS*) (C), *homoglutathione synthetase* (*hGSHS*) (D), were analyzed in uninfected roots and galls. Graphs show gene expression in galls as a multiple of that in uninfected roots. Data (technical triplicates of three biological experiments) are reported as means ± standard error. * indicates a statistically significant difference (P<0.05).

**Figure 2:**
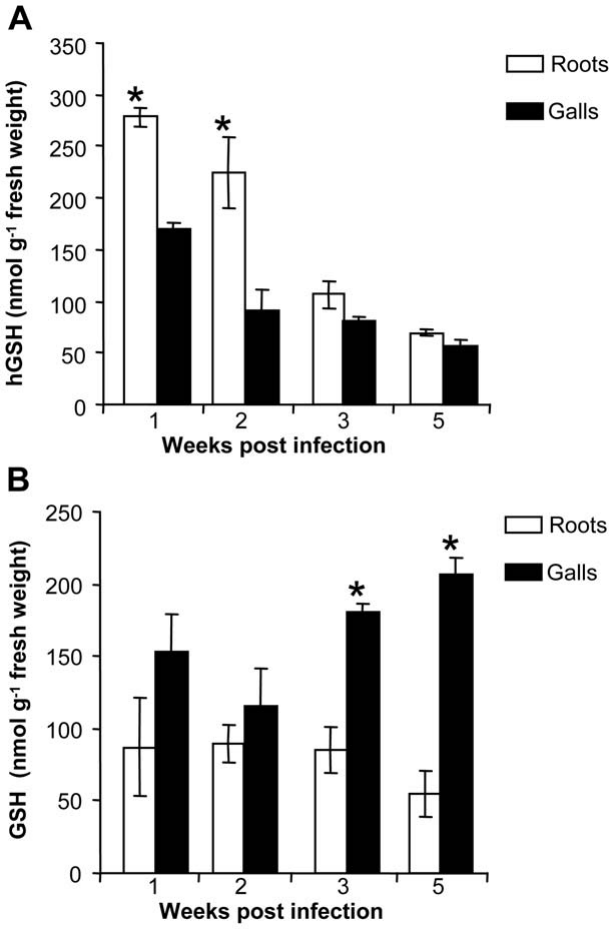
Time course quantification of GSH and hGSH in *M. truncatula* roots and galls. (A) Gall and uninfected root homoglutathione (hGSH) content 1, 2, 3 and 5 weeks post-infection. (B) Gall and uninfected root glutathione (GSH) content 1, 2, 3 and 5 weeks post-infection. The data (6 samples from three independent biological experiments) are reported as mean ± standard error. * indicates a statistically significant difference (P≤0.05).

The development cycle of *M. incognita* in *M. truncatula* is 6–7 weeks long. We analyzed (h)GSH metabolism during the RKN life cycle. First, the expression of *M. truncatula* γ*ECS*, *GSHS* and *hGSHS* genes was evaluated by qRT-PCR (Figure 1A). The expression of γ*ECS* and *hGSHS* was significantly lower in galls than in uninfected roots from 2 wpi (Figure 1B and D). In contrast, no significant difference in the expression of *GSHS* was observed between galls and uninfected roots (Figure 1C). We tested whether the changes in the expression of the genes involved in (h)GSH synthesis correlated with the GSH and hGSH pools (Figure 2A). The quantification of (h)GSH pools by HPLC analysis (Figure 2) showed that hGSH was significantly less abundant in galls than in uninfected roots during the first two wpi corresponding to the period of GC formation (Figure 2A). By contrast, the GSH pool was significantly larger in galls than in uninfected roots 3 and 5 weeks post infection (wpi) with 4 fold-higher level in mature galls 5 wpi (Figure 2B).

### (h)GSH deficiency impairs nematode reproduction and development

**Figure 3:**
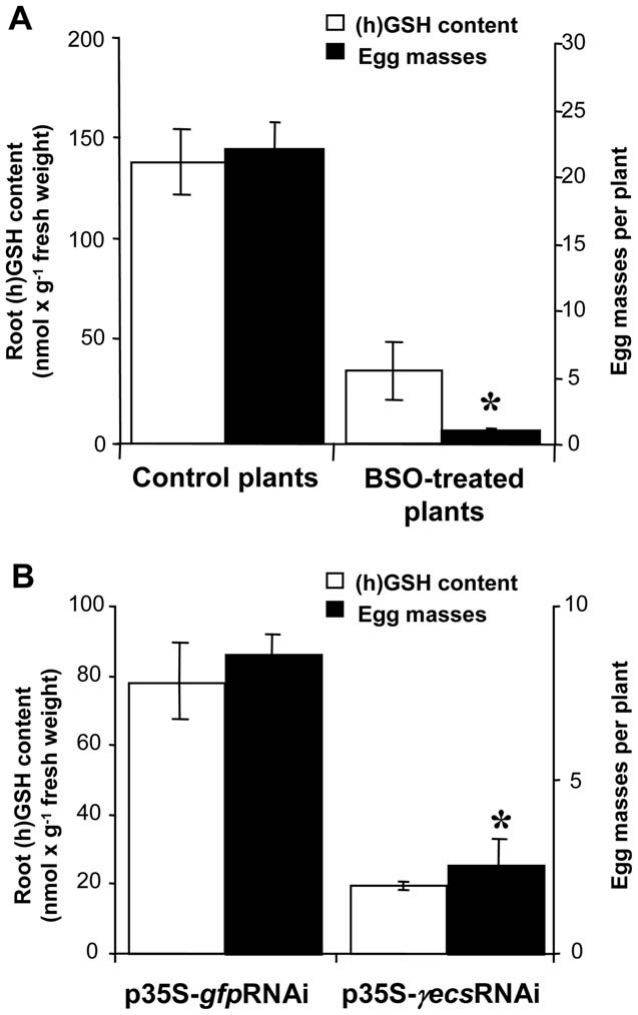
Quantification of (h)GSH and egg mass production in (h)GSH-depleted plants. (h)GSH and egg mass production were quantified in BSO-treated (A) and γECS-RNAi (B). Data (30 plants from three different biological experiments) are reported as mean ± standard error. * indicates a statistically significant difference (P≤0.05).

To assess the involvement of (h)GSH in the plant-nematode interaction, we analyzed the production of egg masses by the nematode in (h)GSH-depleted plants. The plant (h)GSH pool was depleted pharmacologically with L-buthionine-[S–R]-sulfoximine (BSO), a specific inhibitor of (h)GSH synthesis. The effect of BSO treatment on nematode fitness was analyzed by treatment with 1 mM BSO supplemented with 1% resorcinol, a compound shown to induce solute uptake in nematodes [24]. No difference in nematode reproduction was observed between BSO-treated nematodes and controls (Figure S1). Treatment with 0.1 mM BSO applied one week before infection led to an 85% reduction of total (h)GSH in roots as previously described [11]. The primary root of each control and (h)GSH-depleted plants was then inoculated with *M. incognita* and the production of egg masses at 7 wpi was used as a measure of nematode reproduction efficiency (Figure 3). A mean of 23 egg masses was produced in control plants at 7 wpi (Figure 3A). BSO treatment led to a 75% reduction in the (h)GSH content and a 95% diminution of egg mass production in (h)GSH-depleted plants relative to control plants.

To verify that this reduction in egg mass production was related to the decrease in (h)GSH content and not to another secondary effect of BSO, RNAi was used to deplete (h)GSH in *M. truncatula* roots of composite plants [25]. The transgenic roots carrying the γ*ecs*-RNAi construct were compared with transgenic roots expressing an RNAi construct for the Green Fluorescent Protein (GFP) as a control. The number of egg masses and the (h)GSH content of composite plants were analyzed for each individual root at 7 wpi (Figure 3B). Both (h)GSH content and the number of egg masses were significantly lower in the γ*ecs*-RNAi roots than the control *gfp*-RNAi plants. These experiments demonstrate that the reduction in (h)GSH content in galls correlates with a decrease in nematode egg mass production.

**Figure 4:**
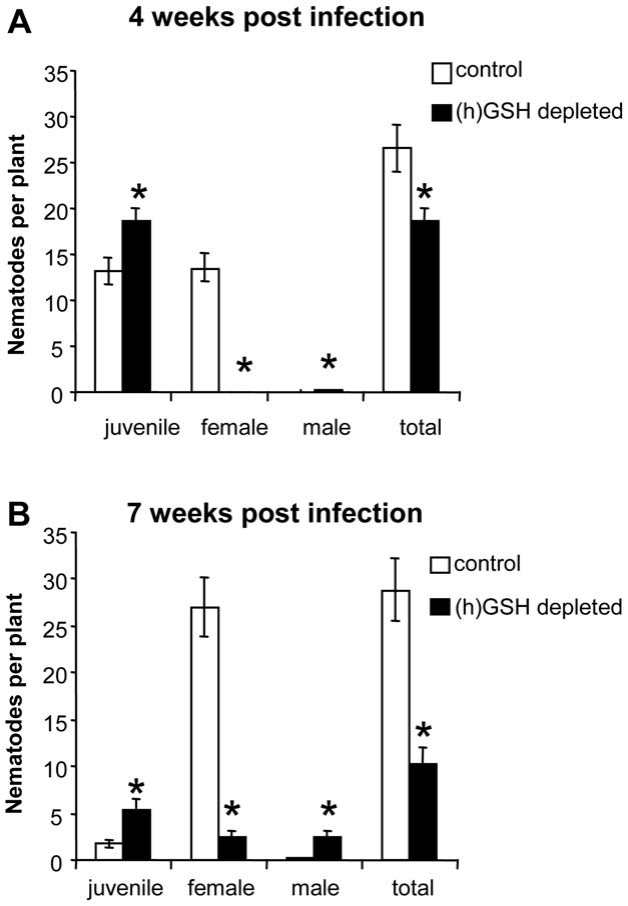
Analysis of nematode developmental stages in (h)GSH-depleted roots. Galls were dissected 4 (A) and 7 (B) weeks post infection and nematode developmental stage (juveniles, female and male) was analyzed. Data (nematodes from 15 plants produced in three different biological experiments) are represented by mean ± standard error. * indicates statistical difference (P<0.05).

As pharmacological and genetic (h)GSH depletion resulted in similarly impaired nematode reproduction, we mainly used BSO treatment to produce sufficient amounts of homogeneous material for further experiments. However, the major results of (h)GSH depletion were confirmed on genetically-modified material. To determine whether the reduction of egg masses was linked to a delay or an arrest in nematode development, galls were dissected at 4 and 7 wpi and the number of nematodes at each developmental stage (juvenile, male and female) was counted (Figure 4). At 4 wpi, an average number of 27 nematodes were detected per control plant whereas only 18 nematodes were observed in each (h)GSH-depleted root, suggesting that nematode infection is affected by (h)GSH depletion. Thirteen of the nematodes were at the female stage in control galls. In contrast, no female was observed in (h)GSH-depleted galls: almost all nematodes were at the juvenile stage and few males were identified in (h)GSH-depleted galls (Figure 4). Under genetic (h)GSH depletion, a significant lower number of nematodes at the female stage was also observed in the γ*ecs*-RNAi roots than in the *gfp*-RNAi control ones (Figure S2A). Moreover, the proportion of males was also significantly increased in γ*ecs*-RNAi roots (Figure S2B). At 7 wpi, the proportion of females was much lower in (h)GSH-depleted galls (23%) than in control galls (90%) and more than half of the nematodes were still juvenile; (h)GSH-depleted galls also contained a large proportion of males (23% vs 0.1% in controls) (Figure 4). Thus, the (h)GSH depletion substantially reduced the number of females per gall (from 27 for controls to 2.3 for (h)GSH-depleted galls) consistent with its effects on egg mass formation. In addition, the numbers of nematodes in (h)GSH-depleted galls at 7 wpi shows that juveniles present at 4 wpi mostly molted into males, or were eliminated from the gall.

### (h)GSH deficiency does not alter giant cell ontogenesis

**Figure 5:**
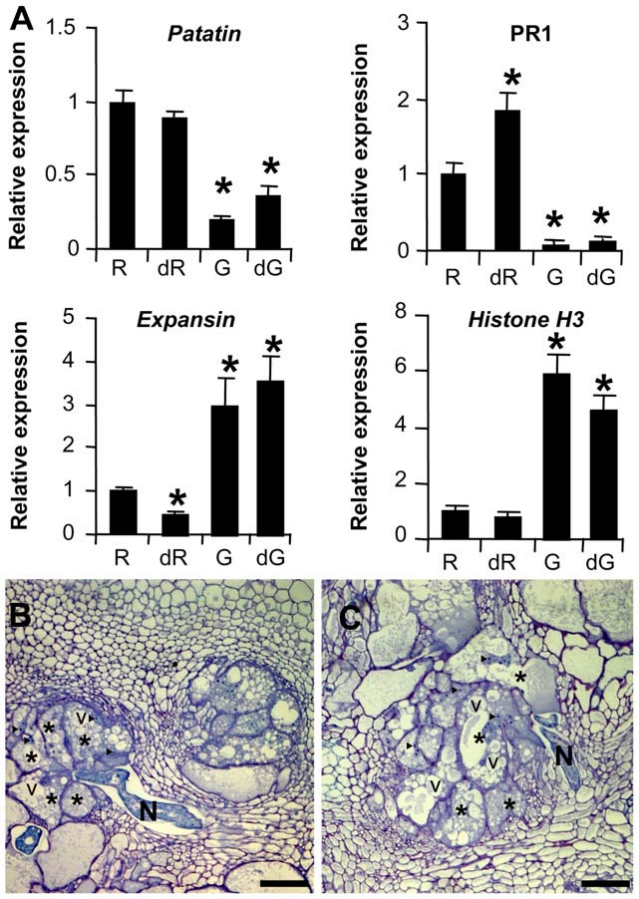
qRT-PCR expression analysis of gall marker genes in control and (h)GSH-depleted roots/galls. (A)The expression levels of the *M. truncatula Patatine*, *Pathogenesis Related Protein 1* (PR1), *Expansin* and *Histone H3* were analyzed 2 weeks post infection in uninfected roots (R), (h)GSH-depleted uninfected roots (dR), galls (G) and (h)GSH-depleted galls (dG). Graphs show gene expression for galls as a multiple of that in uninfected roots. Data (technical triplicates of three biological experiments) are given as means ± standard error. * indicates a statistically significant difference (P<0.05) between uninfected roots and the other samples. (B) and (C) Cross sections at 2 weeks post infection through control gall (B) and (h)GSH-depleted gall (C). Asterisks, giant cells; N, nematode; v, vacuole; ▸, nucleus. Bars  =  100 µm.

The dissection analysis showed that nematode development was impaired at four wpi as no female was detected in (h)GSH-depleted plants at this time point. To test the effect of (h)GSH deficiency on the formation of GC, molecular and cellular analyses were performed at 2 wpi (Figure 5). First, the expression of marker genes involved in GC development was evaluated by qRT-PCR (Figure 5A). The establishment of RKN infestation is associated with the suppression of plant defense responses and the induction of genes encoding proteins involved in both cell wall and DNA metabolism [22]. We therefore studied the expression of the defense-related genes, *Pathogenesis-Related 1 protein* and *patatin*, of *expansin* and of *histone H3*. During the interaction between *M. truncatula* and *M. incognita*, the expression of both *Pathogenesis-Related 1 protein* and *patatin* was significantly weaker in galls at 2 wpi than in uninfected controls; however, the expression of both *expansin* and *histone* was higher in galls than controls. No significant difference was observed between the expression of these four marker genes in (h)GSH-depleted galls and that in the controls. GC morphology was analyzed to detect potential morphological effects (Figures 5B and 5C). Microscopic analysis at 2 and 3 wpi revealed GCs with dense cytoplasm, multiple small vacuoles and nuclei observed in both (h)GSH-depleted galls and control galls (Figures 5B, 5C and Figure S3). Thus, there was no significant molecular or cellular difference between (h)GSH-depleted galls and control galls strongly suggesting that GC ontogenesis was unaffected by (h)GSH depletion.

### Characterization of gall metabolism

**Figure 6:**
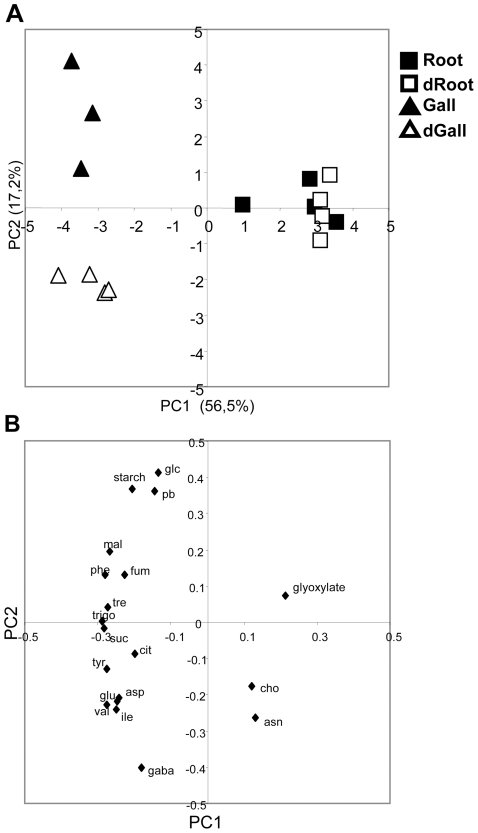
PCA analysis of the absolute concentration of metabolites in control and (h)GSH-depleted roots and galls. (A) PCA scores plot of absolute concentration of metabolites in control roots (Root), (h)GSH-depleted roots (dRoot), galls (Gall) and (h)GSH-depleted galls (dGall) from 3 or 4 biological experiments. (B) PCA loadings plot. For each principal component, loadings are indexed with the corresponding metabolite names. Glucose (glc), sucrose (suc), trehalose (tre), citrate (cit), fumarate (fum), malate (mal), asparagines (asn), aspartate (asp), γ*-*amino butyrate (gaba), glutamate (glu), isoleucine (ile), phenylalanine (phe), tyrosine (tyr), valine (val), trigonelline (trigo), choline (cho), glyoxylate, proline betaine (pb), starch.

Depletion of (h)GSH was thus associated with impaired nematode development and in particular the absence of females at 4 wpi. We performed a metabolomic analysis of control and (h)GSH-depleted roots and galls at 3 wpi to assess primary metabolic effects. We investigated the major compounds of the primary metabolism in roots and galls through an untargeted proton Nuclear Magnetic Resonance (^1^H-NMR*)* analysis approach. Eighteen primary and secondary metabolites were identified in the ^1^H-NMR spectra of each extract (Figure S4) after peak assignment using chemical shift reported in the literature and metabolomic databases, with assistance from 2D NMR and/or by spiking samples with commercial compounds. Eighteen additional metabolites remained unidentified. Clear differences between uninfected roots and galls were obvious on visual inspection of spectra and were confirmed by quantification of metabolites. Eighteen of the 37 quantified metabolites were related to sugar, organic acid and amino acid metabolism (Table 1). Starch, an important sugar reservoir in nematode-induced syncytia [26], was assayed enzymatically. Principal component analysis (PCA) was used to provide an overview of sample grouping and metabolic differences between uninfected roots and galls: we used a matrix containing the data for the 18 identified and quantified polar metabolites plus starch (Figure 6). The first principal component (PC1) of the score plots (Figure 6A), explaining 56% of the total variability, clearly separated galls (on the negative side) from uninfected roots (on the positive side). The loading analysis (Figure 6B) suggested that the major metabolites contributing to this separation along PC1 were sucrose, trehalose, malate, fumarate, six amino acids (aspartate (Asp), glutamate (Glu), isoleucine (Ile), phenylalanine (Phe), tyrosine (Tyr) and valine (Val)) and trigonelline on the negative side and glyoxylate on the positive side. PCA score plot also showed that the second principal component (PC2), explaining 17% of the total variability, clearly separated (h)GSH-depleted (on the negative side) from control galls (on the positive side) (Figure 6A). Observation of PC2 loading (Figure 6B) suggested that this separation along PC2 mainly involved γ-aminobutyrate (GABA), Ile, Val, Glu, Asp and Asn on the negative side and glucose, starch and proline-betaine on the positive side.

**Table 1:** Mean concentration of individual metabolites in roots, (h)GSH-depleted roots (Depleted root), galls and (h)GSH-depleted galls (Depleted gall).

	Quantification µmol/g DW			
	Root	Depleted root	Gall	Depleted gall
glucose	5.3±1.5	4.7±1.9	18.9±1.1 ^a^	6.3±3.3 ^b^
sucrose	48.9±9.3	52.4±2.4	124.5± 9.8 ^a^	154.3±21.5 ^a^
trehalose	1.8±0.1	1.6±0.6	5.3±1.0 ^a^	4.6±0.4 ^a^
citrate	18.9±9.0	14.2±1.4	24.3± 2.8	26.8±2.3
fumarate	1.2±0.1 b	1.1±0.1 b	1.7±0.1 ^a^	1.4±0.2 b
malate	13.4±7.0	11.8±1.4	55.9±6.0 ^a^	33.3±5.7 ^a b^
asparagine	160.1±30.4	115.5±31.9	54.7±20.7 ^a^	122.6±30.0
aspartate	3.4±0.7	5.0±1.3	7.8±0.9 ^a^	11.0±1.3 ^a b^
GABA	nd	nd	nd	4.5±0.4 ^a b^
glutamate	34.2±1.8	30.5±2.7	41.6±2.1 ^a^	53.2±3.8 ^a b^
isoleucine	1.4±0.4	0.6±0.2 ^a^	2.4±0.8	3.7±0.5 ^a^
phenylalanine	3.3±0.3	2.3±0.1 ^a^	8.6±0.7 ^a^	6.3±0.0 ^a b^
tyrosine	0.6±0.1	0.9±0.1 ^a^	1.7±0.1 ^a^	2.1±0.0 ^a b^
valine	1.7±0.2	1.4±0.1	3.3±0.5 ^a^	4.7±0.2 ^a b^
trigonelline	1.3±0.1	2.0±0.1 ^a^	6.0±0.5 ^a^	5.9±0.3 ^a^
choline	5.9±1.3	7.6±1.5	5.1±1.1	5.7±0.6
glyoxylate	3.2±1.0	6.8±2.4	0.9±0.2 ^a^	0.7±0.6 ^a^
proline-betaine	3.0±0.3	3.8±0.5	48.8±23.9 ^a^	8.6±1.5 ^a^
starch	18.4±3.7	4.7±0.8 ^a^	52.6±5.1 ^a^	17.9±3.7 ^b^

Mean of 3 to 4 replicates ±standard error. ^a^ indicates statistical difference (P<0.05) between uninfected roots and the other tissues. ^b^ indicates a statistical difference (P<0.05) between galls and (h)GSH-depleted galls.

**Figure 7:**
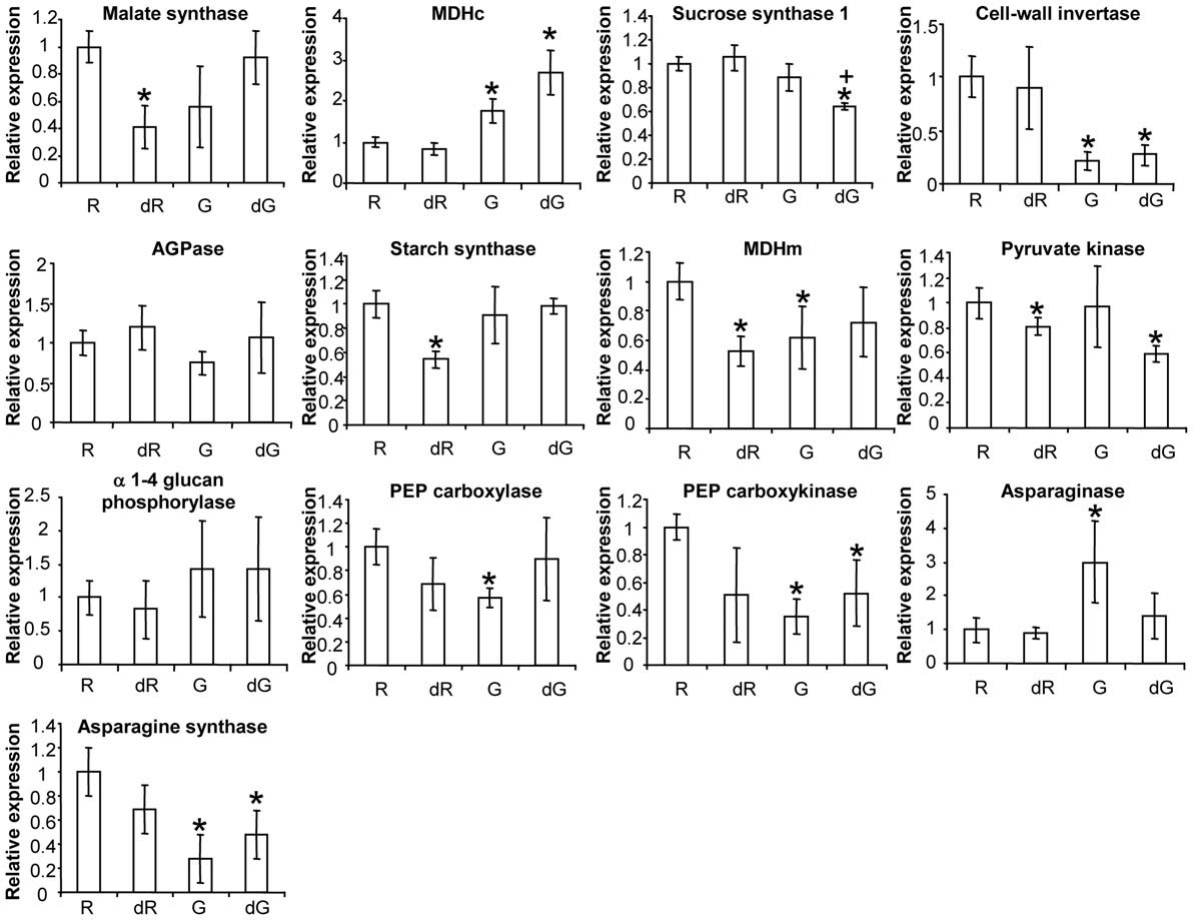
qRT-PCR expression analysis of genes involved in primary metabolism in control and (h)GSH-depleted roots/galls. The expression levels of the *malate synthase, cytosolic malate dehydrogenase (MDHc), sucrose synthase 1, cell wall invertase, ADP-glucose pyrophosphorylase (AGPase), starch synthase, mitochondrial malate dehydrogenase, pyruvate kinase,* α *1-4 glucan phosphorylase, phosphoenolpyruvate (PEP) carboxylase, phosphoenolpyruvate (PEP) carboxykinase asparaginase, asparagine synthase* genes were analyzed 3 weeks post infection in uninfected roots (R), (h)GSH-depleted uninfected roots (dR), galls (G) and (h)GSH-depleted galls (dG). Data (technical triplicates of three biological samples) are reported as means ± standard error. * indicates a statistically significant difference (P≤0.05) between uninfected roots and the other samples. + indicates a statistically significant difference (P≤0.05) between galls and (h)GSH-depleted galls.

**Figure 8:**
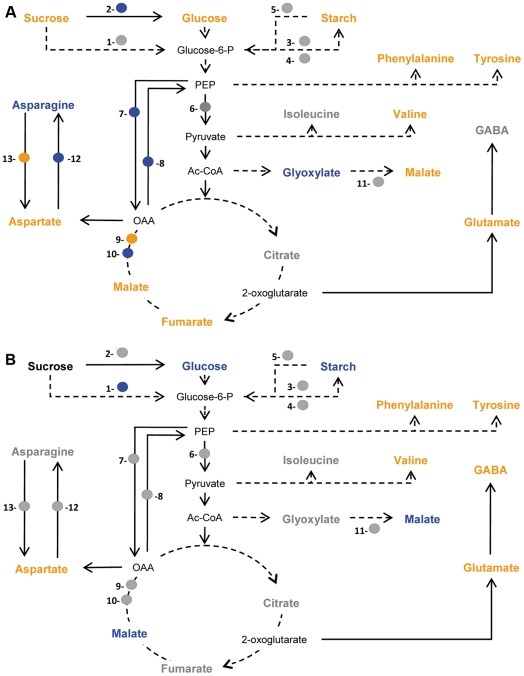
Metabolic changes in control and (h)GSH-depleted galls. Metabolites shown in bold were quantified. The expression levels of the *sucrose synthase 1* (1), *cell wall invertase* (2), *ADP-glucose pyrophosphorylase* (3), *starch synthase* (4), α *1*–*4 glucan phosphorylase* (5), *pyruvate kinase* (6), *phosphoenolpyruvate carboxylase* (7), *phosphoenolpyruvate carboxykinase* (8), *cytosolic malate dehydrogenase* (9), *mitochondrial malate dehydrogenase* (10), *malate synthase* (11), *asparagine synthase* (12) *asparaginase* (13), genes was analyzed. (A) Gall data were normalized with respect to the mean response calculated from the data for uninfected roots. (B) (h)GSH-depleted gall data were normalized with respect to the mean response calculated from data for galls. A color code indicates that values for metabolite content were significantly different from those in control material as assessed by a t-test analysis (P≤0.05). Values for metabolites and genes shown in blue were lower than those for controls and those shown in orange were higher.

The PCA was confirmed by univariate analyses of metabolite data (Table 1). Relative to control roots, galls exhibited a significantly higher content of starch, sugars (sucrose, glucose), organic acids (malate, fumarate) and amino acids (Phe, Tyr, Val, Glu, Asp). However, the increase in amounts of these metabolites was not related to a similar modification of the expression of primary metabolism genes, the expression of which was maintained (*Sucrose synthase 1, ADP-glucose pyrophosphorylase, starch synthase,* α *1*–*4 glucan phosphorylase, pyruvate kinase*) or even decreased (*cell wall-invertase, mitochondrial malate dehydrogenase, malate synthase, phosphoenolpyruvate carboxylase, phosphoenolpyruvate carboxykinase*) in galls (Figure 7). Interestingly, the asparagine (Asn) content of control galls was significantly lower than that of control roots (Table 1). This was related to a significant decrease in expression of the *asparagine synthetase* and an increase in that of *asparaginase* (Figure 7). Proline-betaine, production of which in plants is related to the water stress response, accumulated significantly more in galls than control roots (Table 1). Trigonelline, another aminated compound related to secondary metabolism and potentially involved in salt-stress response [27], was also more abundant in galls than control roots (Table 1). Finally, trehalose accumulation may also be related to a modification of the osmotic status in galls [28]. We used these metabolite and gene expression data to establish a metabolic pathway scaffold (sucrose and starch metabolism, glycolysis and the tricarboxylic cycle connected branch points toward organic acid and amino acid synthesis) highlighting the significant differences observed between roots and galls (Figure 8A). Generally, there is little correlation between increased accumulation of quantified metabolites and the expression of the associated primary metabolism genes.

### (h)GSH deficiency impairs gall metabolism

Depletion of (h)GSH modified the metabolism of roots and galls in different ways. Most metabolites in roots were not significantly modified by (h)GSH-depletion. PCA analysis of the 18 identified and quantified polar metabolites plus starch showed that (h)GSH-depleted and control uninfected roots had a similar composition of polar metabolites (Figure 6A). Significant variations were observed only for the hydrophobic amino acids Ile, Phe and Tyr, for trigonelline and for starch (Table 1). Indeed, the starch content was decreased 3-fold and *starch synthase* was significantly down regulated by (h)GSH depletion, suggesting that starch metabolism in roots is regulated by (h)GSH content or metabolism. Unlike the findings for uninfected roots, (h)GSH depletion had substantial effects on the metabolism of galls (Figure 6). The content of nine metabolites differed significantly between (h)GSH-depleted galls and control galls (Table 1). Starch and glucose contents were significantly lower in (h)GSH-depleted galls, whereas that of sucrose was not significantly different. With the exception of malate, the abundance of which was significantly decreased, the organic acid content of galls was not significantly affected by (h)GSH depletion. In the γ*ecs*-RNAi galls, starch, glucose and malate contents were also significantly lower than in the control *gfp*-RNAi galls (Table S1). Consistent with these findings, the relative expression in galls of most genes involved in the metabolism of sugars and organic acids was not significantly modified by (h)GSH depletion. The amino acids content was slightly increased by (h)GSH depletion, with the exception of Asn which was increased two-fold to the range of that found in roots (Table 1). This increase in Asn content was associated with a significant decrease in *asparaginase* gene expression and a two-fold increase in *asparagine synthetase* gene expression associated with (h)GSH depletion (Figure 7). A similar trend was also observed for proline-betaine, the content of which in (h)GSH-depleted galls was close to that in uninfected roots. Thus, our data indicate that (h)GSH depletion partially reversed the effect of nematode infection on starch, glucose and Asn metabolism, and on proline-betaine accumulation. Interestingly, GABA, a compound associated with biotic and abiotic stresses [29], was markedly more abundant in (h)GSH-depleted than control galls. The significant differences between control and (h)GSH-depleted galls are summarized in a metabolic pathway scaffold (Figure 8B). As observed for the comparison between galls and uninfected roots, differences in metabolite contents were more marked than the differences between gene expression levels. This implies post-transcriptional regulatory mechanisms, such as post-translational modifications or metabolic controls, in the metabolic modifications associated with (h)GSH depletion of galls.

## Discussion

(h)GSH play a major role in plant development and plant adaptation to biotic and abiotic stresses [30]. A threshold (h)GSH concentration is necessary for plant and organ development [5]–[7], [31], [32]. (h)GSH is also involved in plant responses to pathogens [2], [3] and to symbiotic microorganisms [11]. Here, we report an analysis of the involvement and roles of (h)GSH metabolism in the *M. truncatula*-*M. incognita* interaction.

### The gall is characterized by an adapted (h)GSH metabolism

*M. truncatula* roots contain two low molecular weight thiols, GSH and hGSH [9]. The (h)GSH content is significantly higher in galls than in roots at later stages of gall functioning. Surprisingly, γ*ECS* transcript level was lower in galls than in roots whereas this gene should regulate the level of (h)GSH. The post-transcriptional regulation of γECS [33], [34] may explain the discrepancy between γ*ECS* transcript level and GSH accumulation. (h)GSH accumulation has been observed in several developmental conditions involving endoreduplication and enhanced metabolic capacity such as in symbiotic nitrogen fixation [9] and in trichomes [35], both physiological modifications also occurring during gall formation and function [18], [22], [36]. In addition, the accumulation of GSH in galls may be caused by the nematode, as a GSHS has been identified amongst the proteins secreted by *M. incognita* [37]. Finally, (h)GSH accumulation is also associated with the nematode secreting multiple redox- and (h)GSH-regulated proteins, including thioredoxin, glutathione peroxidases and glutathione-S-transferases, required for the completion of nematode life cycle [37], [38]. Indeed, the control of the plant cell redox status through the modification of the (h)GSH content may be a key regulator of the GC effectiveness.

### (h)GSH deficiency does not affect development of the feeding site but impairs nematode reproduction and development

We show here that root (h)GSH deficiency strongly impairs nematode reproduction. This reduction of egg masses seems to be largely a consequence of the nematode sex ratio in galls. At 7 wpi, galls in (h)GSH-depleted plants harbored only one third as many nematodes as controls, suggesting that most juveniles developed into males and therefore migrated from the gall to soil such that they were not found in the gall by dissection. An hypothesis might be that BSO would be involved in direct impairment of GSH production in nematodes [39], [40] and thus modify their development and egg mass production. Analysis of *M. incognita* genome using *Caenorhabditis elegans* γ*ECS* and *GSHS* sequences shows that the GSH biosynthesis genes are present in *M. incognita.* Moreover, HPLC analysis shows that GSH is produced in *M. incognita* J2 larvae (unpublished data). The effect of GSH depletion on *M. incognita* development could not be directly tested as it is a plant obligatory parasite. However, data provided on WormBase (http://www.wormbase.org) showed that GSH does not play a major role in both development and health in *C. Elegans.* GSH depletion induced by γ*ECS-*RNAi or gene deletion is not larval or embryonic lethal and does not induce slow growth and female sterility [41]–[43]. Finally, the lower egg mass production, the modifications of the nematode sex-ratio and metabolite contents observed in both BSO-treated plants and transgenic roots expressing a plant specific γ*ECS*-RNAi construct show that these modifications are not linked to direct impairment of nematode function by BSO.

During symbiosis between *M. truncatula* and *S. meliloti*, the (h)GSH depletion reduces the formation of nodule meristems [11]. Transcriptomic analysis evidences the involvement of (h)GSH regulation both in plant development and defense responses [12]. In contrast, under similar conditions, the development of the feeding site was not significantly affected and the expression of defense-related and development-related genes was not modified. Therefore, *M. incognita* is able to manipulate plant metabolism under (h)GSH depletion to avoid the defense and developmental phenotype observed during the establishment of nitrogen-fixing symbiosis.

### The impairment of nematode development and reproduction in (h)GSH-depleted galls is linked to a modified carbon metabolism at the feeding site

Root and gall metabolomic profiling showed that most of the analyzed metabolites were significantly more abundant in galls than in uninfected roots. These modifications, and the analysis of the expression of numerous genes involved in primary metabolism, indicate that the gall metabolism differs substantially from that in uninfected roots. One of the striking differences concerning general metabolite accumulation is the significantly lower Asn content in galls than in roots. This, and the associated upregulation of *asparaginase* and the down regulation of *asparagine synthase,* shows that nitrogen metabolism is modified in galls. Asn is the major nitrogen transporting compound in temperate legumes such as *Medicago* [44], [45]. The primary site of Asn synthesis is the root and it follows that, through loading into the xylem, Asn is the principal nitrogen source for amino acids and protein synthesis in leaves. Thus, the decrease in Asn content upon nematode infection is likely to result in nitrogen deprivation for the plant. This metabolic modification may thus reduce nitrogen supply to leaves and increase carbon and nitrogen accumulation in galls. Our findings show that gall metabolism involves the fine-tuning of metabolism involving both the up regulation of some metabolic pathways and the down regulation of others, so as to enhance nutriment availability for the nematodes.

Analysis of metabolite contents shows that (h)GSH depletion significantly affect gall metabolism. A significant difference in metabolite content between control and (h)GSH-depleted galls was detected for half of the metabolites quantified. In contrast, (h)GSH depletion did not significantly modify the content of the major primary metabolites in uninfected roots. This result is in agreement with our previous findings that a 85% depletion of (h)GSH does not significantly affect root growth [11]. The metabolic modifications observed in (h)GSH-depleted galls include a significant reduction of malate (40%), glucose (60%) and starch (84%). Starch accumulation during the interaction between *A. thaliana* and the parasitic nematode *Heterodera schachtii* is crucial for the nematode infection and development. It may serve as long- and short-term carbohydrate storage for the feeding needs of the parasites [26]. Glucose and malate are likely substrates and probably essential for nematode nutrition. Thus, the diminution of these three metabolites under (h)GSH depletion may impair nematode carbohydrate nutrition. The development of *M. incognita* juveniles into males rather than females has previously been observed under unfavorable nematode feeding conditions such as low concentrations of sucrose in the growth medium, defoliation and complete removal of the host plant above-ground parts [46]–[48]. These conditions also trigger carbon starvation of the galls. Carbohydrate nutrition deficiency has been also involved in the modification of sex ratio and development of cyst nematode [49]. The development of *M. incognita* juveniles into males rather than females in (h)GSH-depleted galls is similar to that observed during carbohydrate deficiency. This is consistent with (h)GSH being involved in the modulation of nematode differentiation through regulation of gall carbon metabolism.

Interestingly, GABA was specifically detected in (h)GSH-depleted galls. In plants, GABA accumulates in response to abiotic and biotic stresses [50]. During biotic stress induced by invertebrate pests, GABA accumulation in plant tissues reduces the feeding capacity of the pests [51]. Strikingly, the reproduction of *Meloidogyne hapla* is affected by GABA accumulation: egg mass production by *M. hapla* infecting transgenic plants accumulating GABA is lower than that by the pests infecting control plants [52]. Thus, the accumulation of GABA in (h)GSH-depleted galls may also contribute to the altered nematode reproduction.

The substantial primary metabolite modifications in (h)GSH-depleted galls with reference to control galls were not associated with corresponding modification in the expression of primary metabolism genes. This suggests that (h)GSH regulates gall metabolism at levels other than transcriptional. Redox state and GSH affect the function of many enzymes through post-translational modifications such as disulfide bond reduction and cysteine glutathionylation [53]. For instance, thioredoxins and glutaredoxins, which are involved in the formation/reduction of disulfide bonds between proteins, have been implicated in the regulation of chloroplast metabolism [54], [55]. ADP glucose pyrophosphorylase, a key enzyme in the biosynthesis of starch was also shown to be redox regulated [56], [57]. Cysteine glutathionylation is an important regulatory mechanism of photosynthetic metabolism [58]. More generally, *in vivo* control of many glycolytic and/or TCA cycle enzymes by disulfide-dithiol interconversions (NAD-dependent GAPDH, citrate synthase, PPi-dependent phosphofructokinase, PEPC kinase, etc) has been reported in plants [59]. Thus, a redox-based control of the gall metabolism by (h)GSH may be proposed to explain our results.

We cannot exclude that GSH may also be used as a nutrient by the nematode as the GSH content was strongly increased in mature galls compared to roots. However, the impact of (h)GSH depletion on gall metabolism is not in favour of a trophic role for (h)GSH. Moreover, during nitrogen fixing symbiosis in which GSH is not used as nutrient to feed the bacteroids, modifications of the (h)GSH content affects the nitrogen-fixing capacity of the nodule also showing the regulatory role of glutathione in this interaction [60].

In conclusion, we report that (h)GSH metabolism differs between galls and uninfected roots. A deficiency in (h)GSH impairs nematode reproduction by mainly altering its sex determination. This alteration in sex ratio is associated with modifications in the gall metabolism under (h)GSH depletion which have been shown to impair nematode development. Thus, we reveal a completely new role of (h)GSH in this biotrophic interaction. Interestingly, these modifications in metabolite content do not seem to occur in (h)GSH-depleted roots suggesting that (h)GSH depletion provokes metabolism modifications specific to the gall. Therefore, the reduction of (h)GSH availability in galls is a potentially useful strategy for pest management.

## Materials and Methods

### Plant material, growth condition, treatments and nematode infection

*M. truncatula* ecotype A17 was used for all the experiments. Sterilized seeds were germinated for 3 days onto 0.4% agar at 14°C. Seedlings were plated onto modified Fahraeus medium with 2 mM nitrogen [25] with 1.4% agar and grown for 7 days before infection. Plants were germinated in the presence or absence of 0.1 mM L-buthionine sulfoximine (BSO). For nematode infection, 100 surface-sterilized freshly hatched *M. incognita* J2 larvae were added on each one week old seedling as previously described [13]. One infection per plant was performed on the primary root. For BSO treatment, nematodes were incubated for 4 hours in M9 buffer (43.6 mM Na_2_HPO_4_, 22 mM KH_2_PO_4_, 2.1 mM NaCl, 4.7 mM NH4Cl) with 1% resorcinol and 1 mM BSO and with 1% resorcinol as control. The gall corresponds to one infection point and contains multiple GCs. After infection, plants were grown 3 weeks onto Fahraeus medium with 2 mM nitrogen in the presence or absence of BSO (0.1 mM) in a growth chamber with a day temperature of 23°C and night temperature of 20°C and with a photo-period of 16 h. Then, plants were transferred in soil mixture (30% vermiculite-70% fine gravel) until the end of the experiment. As reference samples, uninfected, primary root fragments of similar age were collected from seedlings grown under the same conditions. For gall dissection, galls were digested in a mixture of 30% Pectinex (Novozymes, Bagsvaerd, Denmark) and 15% Celluclast BG (Novozymes, Bagsvaerd, Denmark) for 12 h, dissected and nematode development stages were analyzed under a stereomicroscope. For metabolite and gene expression analyses, biological samples of galls and roots were harvested at different time points post-infection, frozen and ground in liquid nitrogen and stored at −80°C. One biological sample was issued from 20 galls or roots from 20 plants.

### GSH and hGSH determination

Thiols were extracted with perchloric acid, derivatized with monobromobimane, and quantified after separation on reverse-phase HPLC as described previously [61]. Commercial GSH (Sigma, St. Quentin, France) and γ-EC (Promochem, Molsheim, France) were used as standards. The hGSH used as a standard was synthesized by Neosystem (Strasbourg, France).

### Gene expression analysis by quantitative RT-PCR

Total RNA of galls and uninfected root fragments were reverse-transcribed using the OmniScript cDNA Synthesis Kit (Qiagen, Courtaboeuf, France). Quantitative PCR reactions were performed using a DNA Engine Opticon 2 Continuous Fluorescence Detection system (MJ Research, Waltham, USA) and a qPCR MasterMix Plus for SYBR green I (Eurogentec, Angers, France). In each reaction, 5 µl of 100 fold-diluted cDNA and 0.3 µM primer (sequences used are described in Table S2) were used. The PCR conditions were 50°C for 5 min, 95°C for 10 min, followed by 40 cycles of 95°C for 30 s, 60°C for 1 min. Each reaction was performed in triplicate and the results represented the mean of three independent biological experiments. The specificity of the amplification was confirmed by a single peak in a dissociation curve at the end of the PCR reaction. Data were quantified by using Opticon Monitor 2 (MJ Research, Waltham, USA) and normalized with the 2^−ΔΔCT^ method [62]. Two constitutively expressed genes *Mtc27* (TC106535) and *40S Ribosomal Protein S8* (TC100533) were the endogenous controls [63]. The use of these housekeeping genes were validated by using the GeNorm VBA applet for MS Excel which determines the most stable housekeeping genes from a set of tested genes in a given cDNA sample panel [64]. PCR reactions for each of the three biological replicates were performed in technical triplicate. The absence of genomic DNA contaminations in the RNA samples was tested by PCR analysis of all samples using oligonucleotides bordering an intron in *M. truncatula GSHS* gene.

### Production of γECS RNAi transgenic plant

To generate the γ-ECS-RNAi construct, a 502-bp region was amplified from the cDNA using gene-specific primers (Supplemental Table S2 on line) and cloned into the pDONR207 vector, subcloned in pENTR4 and integrated into the RNAi vector pK7GWIWG2DII,(0) [65]containing kanamycin resistance and the p35S:eGFP for selection and screening. *M. truncatula* plants (A17) were transformed with *A. rhizogenes* containing precedent construct as described previously [25] and transformed roots were selected by resistance to kanamycin and screening of eGFP. Control plants were transformed with *A. rhizogenes* containing the pKGWIWG2DII,(0) vector containing an eGFP DNA fragment to rule out the potential side effects linked to plant transformation or the RNAi vector.

### NMR metabolomic profiling

Polar metabolites were quantified using ^1^H-NMR of polar extracts. For the preparation of extracts and NMR acquisition parameters, special care was taken to allow absolute quantification of individual metabolites. Briefly, polar metabolites were extracted on lyophilized powder (30 mg DW per biological replicate) with an ethanol–water series at 80°C as described previously [66]. The lyophilized extracts were titrated with KOD to pH 6 in 100 mM potassium phosphate buffer in D_2_O and lyophilized again. Each dried titrated extract was solubilized in 0.5 mL D_2_O with (trimethylsilyl)propionic-2,2,3,3-*d4* acid (TSP) sodium salt (0.01% final concentration) for chemical shift calibration and ethylene diamine tetraacetic acid (EDTA) disodium salt (0.5 mM final concentration). ^1^H-NMR spectra were recorded at 500.162 MHz on a Bruker Avance spectrometer (Bruker, Karlsruhe, Germany) using a 5-mm dual ^13^C-^1^H cryoprobe and an electronic reference for quantification [66]. Sixty-four scans of 32 K data points each were acquired with a 90° pulse angle, a 6000 Hz spectral width, a 2.73 s acquisition time and a 25 s recycle delay. Preliminary data processing was conducted with TOPSPIN 1.3 software (Bruker Biospin, Wissembourg, France). The assignments of metabolites in the NMR spectra were made by comparing the proton chemical shifts with literature [66]–[68] or metabolomic database values (MeRy-B 2009, HMDB), by comparison with spectra of authentic compounds recorded under the same solvent conditions and/or by spiking the samples. For assignment purposes, ^1^H-^1^H COSY, ^1^H-^13^C HSQC and ^1^H-^13^C HMBC 2D NMR spectra were acquired for selected samples.

The metabolite concentrations were calculated using AMIX (version 3.9.1, Bruker) and Excel (Microsoft, Redmond, WA, USA) softwares. The metabolites were quantified using the glucose calibration curve and the proton amount corresponding to each resonance for all compounds. The metabolite concentrations were calculated from concentrations in the NMR tube and sample dry weight.

The 15 ^1^H-NMR spectra of the data set were converted into JCAMP-DX format and deposited with associated metadata into the Metabolomics Repository of Bordeaux MeRy-B (http://www.cbib.u-bordeaux2.fr/MERYB/projects/home.php?R=0&project_id=28). 

To explore the metabolite multidimensional data set, we used principal component analysis (PCA) on mean-centered data scaled to unit variance (MATLAB version 7.4.0, the MathWorks Inc, Natick MA).

### Starch and malate measurement

Starch was recovered from the insoluble fraction of the extracts used for polar metabolite extraction after ethanol–water series at 80°C (see above, Moing et al. 2004). Insoluble residues were incubated for 1 h, at 55°C, in a 0.5 ml reaction medium containing 0.1 M sodium acetate, and 1.25 mg amyloglucosidase (Sigma-Aldrich, Saint-Quentin Fallavier, France). Reaction was stopped for 5 min at 100°C. Supernatants were collected and evaporated over night under vacuum. Dry residues were taken up with 0.5 ml of 0.3 M Hepes, pH 7.5, and 30 mM MgSO_4_. Glucose, issued from starch hydrolysis, was measured as followed: 200 to 400 µl of samples were mixed with a reaction medium containing 0.3 M Hepes, pH 7.5, 30 mM MgSO_4_, 2.5 mM ATP, and 2 mM NAD. Initial OD was red at 340 nm. Next, 2 Units of both hexokinase (Sigma-Aldrich, Saint-Quentin Fallavier, France) and glucose-6-phosphate dehydrogenase from *Leuconostoc mesenteroides* (Sigma-Aldrich, Saint-Quentin Fallavier, France) were added, and samples were incubated for 1 h, at room temperature in the dark. Final OD was red at 340 nm. The difference between the final and initial OD was used to calculate the glucose content. Starch was expressed as nmol of glucose equivalent per dry weight unit.

Malate was quantified by ionic chromatography and conductimetry. Separation was performed on an IonPac AS 11 column (4×250 mm, Dionex, Sunnyvale, CA, USA) and a IonPac AG11 guard column (4×50 mm, Dionex) with a NaOH gradient including 16% of methanol. Calibration was performed with commercial standards using gravimetric method.

### Database searches and sequence analysis

DNA sequences were analyzed using BLAST [69] against the databases of the NCBI (http://blast.ncbi.nlm.nih.gov/), MtGI (http://compbio.dfci.harvard.edu/cgi-bin/tgi/gimain.pl?gudb=medicago) and the IMGAG (http://www.medicago.org/genome/). The accession numbers of the genes used in this study are indicated in the Supplemental Table S2.

### Statistical analyses

All the data presented are given as means with the standard error of three or four independent biological experiments. The significance of the results was tested using Student t-test (P value ≤0.05).

## Supporting Information

Figure S1 **Quantification of egg mass production by BSO-treated nematodes.** Egg mass production was quantified in plants infected with resorcinol-treated and resorcinol/BSO-treated nematodes. Data (15 plants from three different biological experiments) are reported as mean ± standard error.
(PPT)

Figure S2 **Analysis of nematode developmental stages in genetically (h)GSH-depleted roots.** (A) Galls were dissected 4 weeks post infection and nematode developmental stage (juveniles, female and male) was analyzed. (B) Galls were dissected 4 weeks post infection and nematode developmental stage (juveniles, female and male) relative amounts were analyzed. Data (nematodes from 15 plants produced in three different biological experiments) are represented by mean ± standard error. * indicates statistical difference (P<0.05).
(PPT)

Figure S3 **Microscopic analysis of control and (h)GSH-depleted galls.** Cross sections at 3 wpi through wild-type galls (A) and (h)GSH-depleted galls (B). Asterisks, giant cells; N, nematode; v, vacuole; ▸, nucleus. Bars  =  100 µm
(PPT)

Figure S4 **Representative 1D 1H 500 MHz NMR spectra of polar extracts of roots or galls from (h)GSH-depleted and control** ***M. truncatula*** **plants.** Galls were harvested 3 weeks after infection with *M. incognita*. (A) Zoom in on the aromatic region (δ 9.25–5.55). (B) Zoom in on the sugar region (δ 4.7–3.25). (C) Zoom in on the alpha anomeric sugar region (δ 5.5–5); Zoom in on the quaternary amine region (δ 3.34–3.04); Zoom in on the aliphatic regions (δ 3.07–2.48) and (δ 2.2–0.8). (h)GSH: homoglutathione and glutathione; BSO: L-buthionine-[S-R]-sulfoximine, a specific inhibitor of (h)GSH synthesis; EDTA: ethylene diamine tetraacetic acid disodium salt; GABA: γ-aminobutyrate; unk: unknown compound.
(PPT)

Table S1 **Mean concentration of individual metabolites in p35S-** ***gfp*** **RNAi and p35S-** γ***ecs*** **RNAi galls.** Mean of 3 replicates ±standard error. * indicates statistical difference (P<0.05) between p35S-*gfp*RNAi and p35S- γ*ecs*RNAi galls.
(DOC)

Table S2 **List of primers used for the construction of the genetic vectors and to quantify the gene expression levels.**
(DOC)
