# Supplementary figures and images for: (Homo)glutathione Deficiency Impairs Root-knot Nematode Development in Medicago truncatula
Source: PLoS Pathog. 2012 Jan 5;8(1):e1002471. doi: 10.1371/journal.ppat.1002471 (PMC3252378; doi:10.1371/journal.ppat.1002471)

## Slide 1
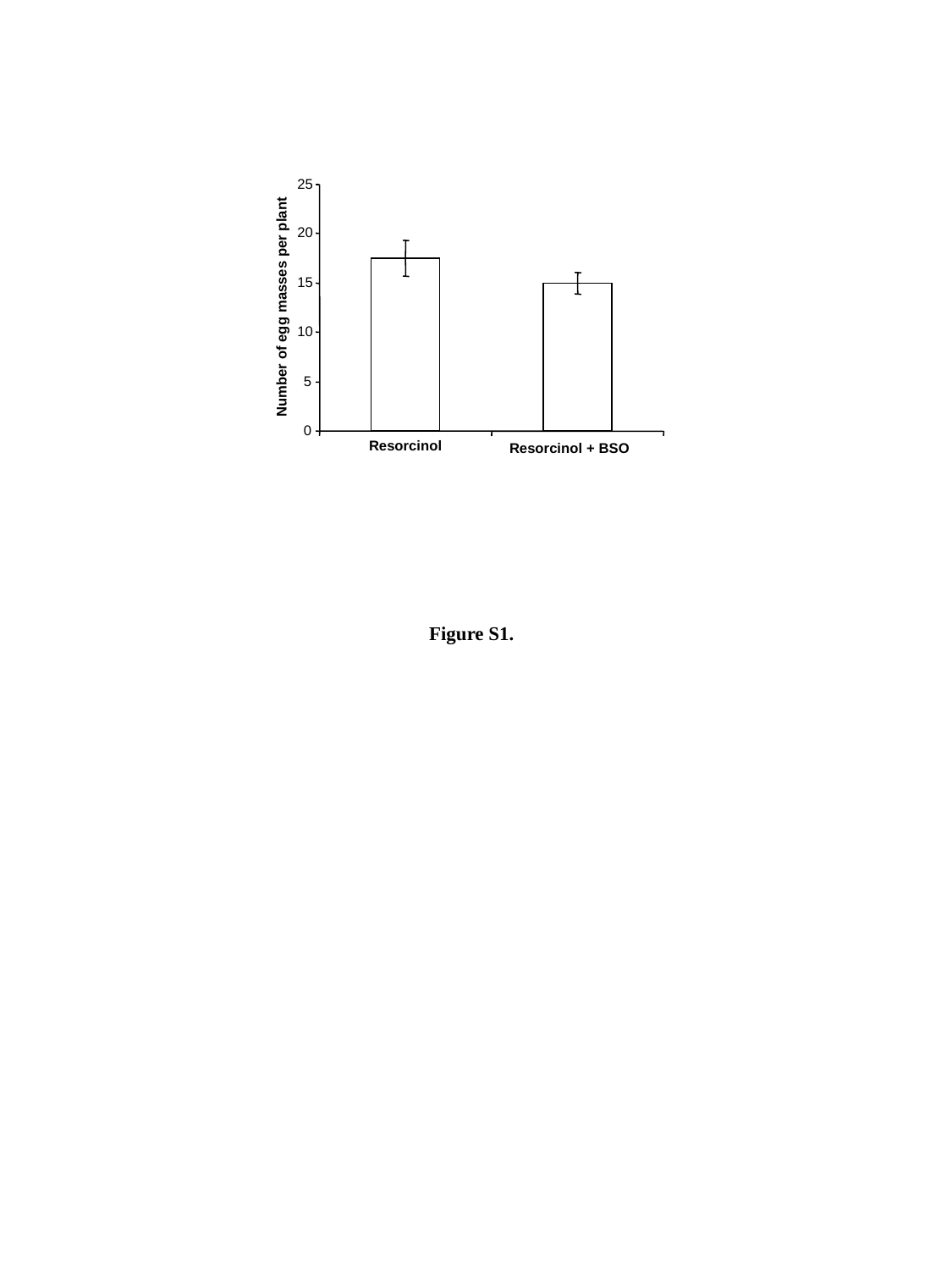

25
20
15
Number of egg masses per plant
10
5
0
Resorcinol
Resorcinol + BSO
Figure S1.

Supplement: Figure S1 — Quantification of egg mass production by BSO-treated nematodes. Egg mass production was quantified in plants infected with resorcinol-treated and resorcinol/BSO-treated nematodes. Data (15 plants from three different biological experiments) are reported as mean ± standard error. (PPT) [file ppat.1002471.s001.ppt]

## Slide 1
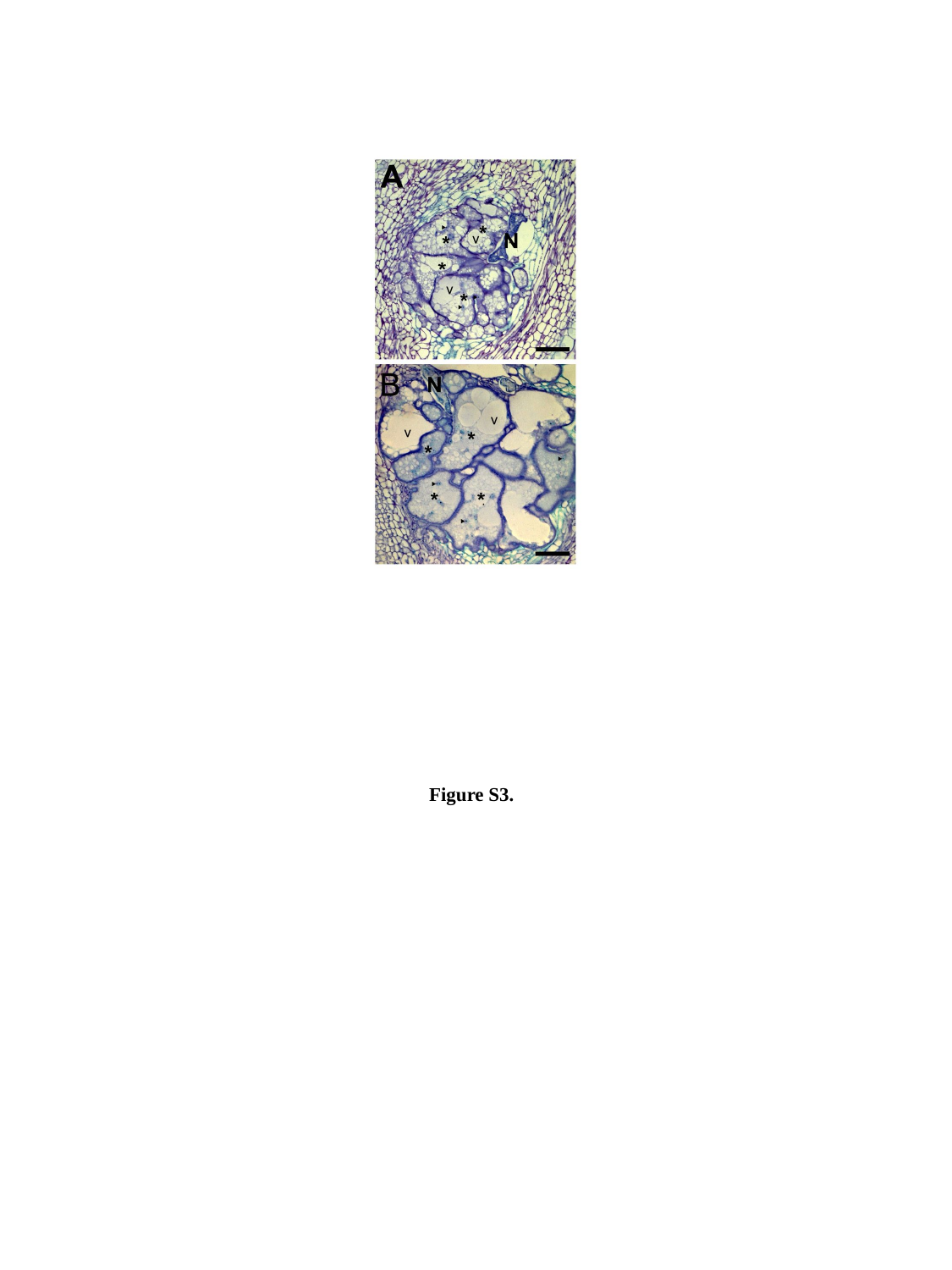

Figure S3.

Supplement: Figure S3 — Microscopic analysis of control and (h)GSH-depleted galls. Cross sections at 3 wpi through wild-type galls (A) and (h)GSH-depleted galls (B). Asterisks, giant cells; N, nematode; v, vacuole; ▸, nucleus. Bars = 100 µm (PPT) [file ppat.1002471.s003.ppt]

## Slide 1
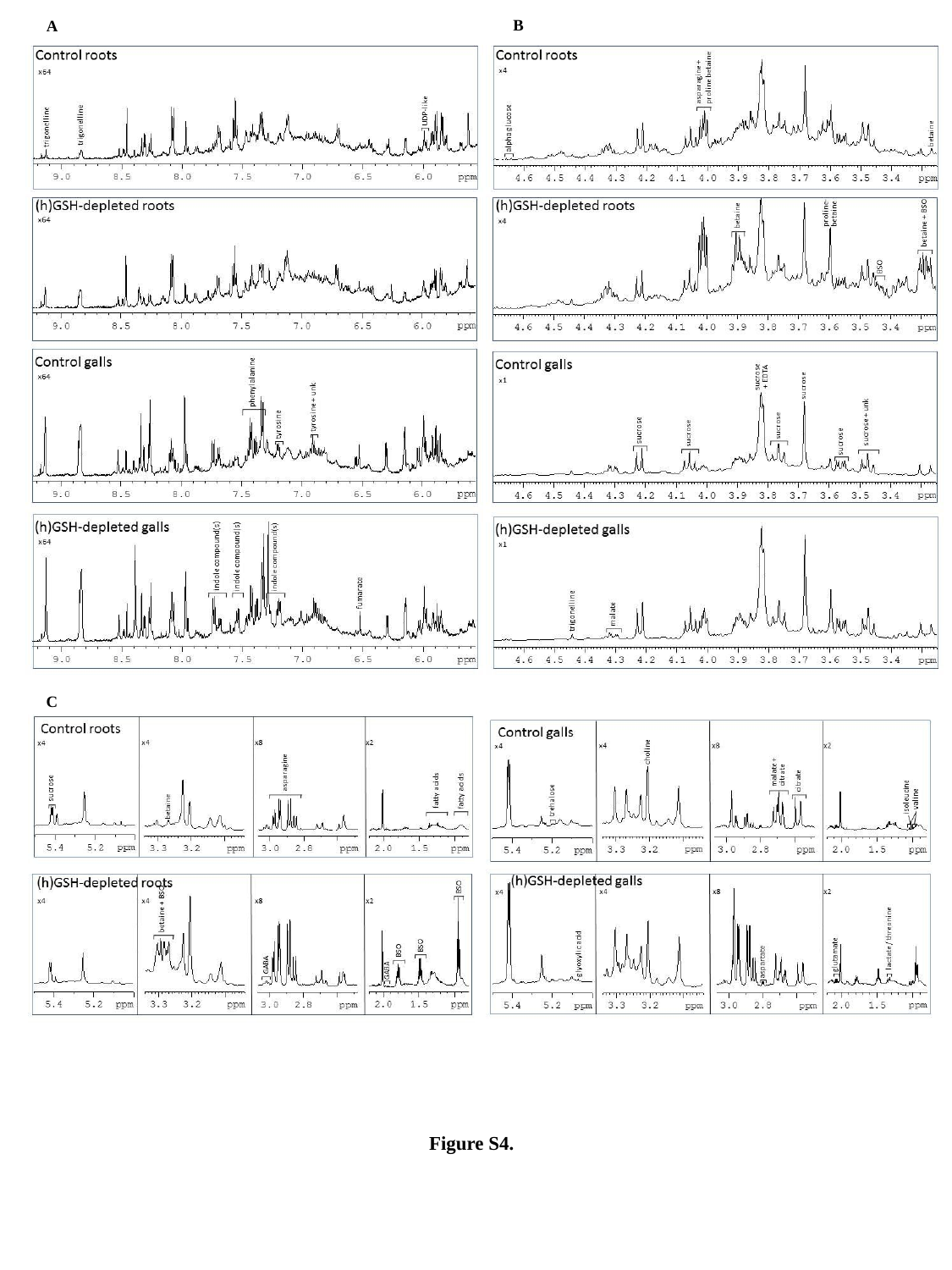

B
 A
 C
Figure S4.

Supplement: Figure S4 — Representative 1D 1H 500 MHz NMR spectra of polar extracts of roots or galls from (h)GSH-depleted and control M. truncatula plants. Galls were harvested 3 weeks after infection with M. incognita. (A) Zoom in on the aromatic region (δ 9.25–5.55). (B) Zoom in on the sugar region (δ 4.7–3.25). (C) Zoom in on the alpha anomeric sugar region (δ 5.5–5); Zoom in on the quaternary amine region (δ 3.34–3.04); Zoom in on the aliphatic regions (δ 3.07–2.48) and (δ 2.2–0.8). (h)GSH: homoglutathione and glutathione; BSO: L-buthionine-[S-R]-sulfoximine, a specific inhibitor of (h)GSH synthesis; EDTA: ethylene diamine tetraacetic acid disodium salt; GABA: γ-aminobutyrate; unk: unknown compound. (PPT) [file ppat.1002471.s004.ppt]
