# Supplementary material for: (Homo)glutathione Deficiency Impairs Root-knot Nematode Development in Medicago truncatula
Source: PLoS Pathog. 2012 Jan 5;8(1):e1002471. doi: 10.1371/journal.ppat.1002471 (PMC3252378; doi:10.1371/journal.ppat.1002471)
Supplement: Figure S2 — Analysis of nematode developmental stages in genetically (h)GSH-depleted roots. (A) Galls were dissected 4 weeks post infection and nematode developmental stage (juveniles, female and male) was analyzed. (B) Galls were dissected 4 weeks post infection and nematode developmental stage (juveniles, female and male) relative amounts were analyzed. Data (nematodes from 15 plants produced in three different biological experiments) are represented by mean ± standard error. * indicates statistical difference (P<0.05). (PPT) [file ppat.1002471.s002.ppt]

## Slide 1
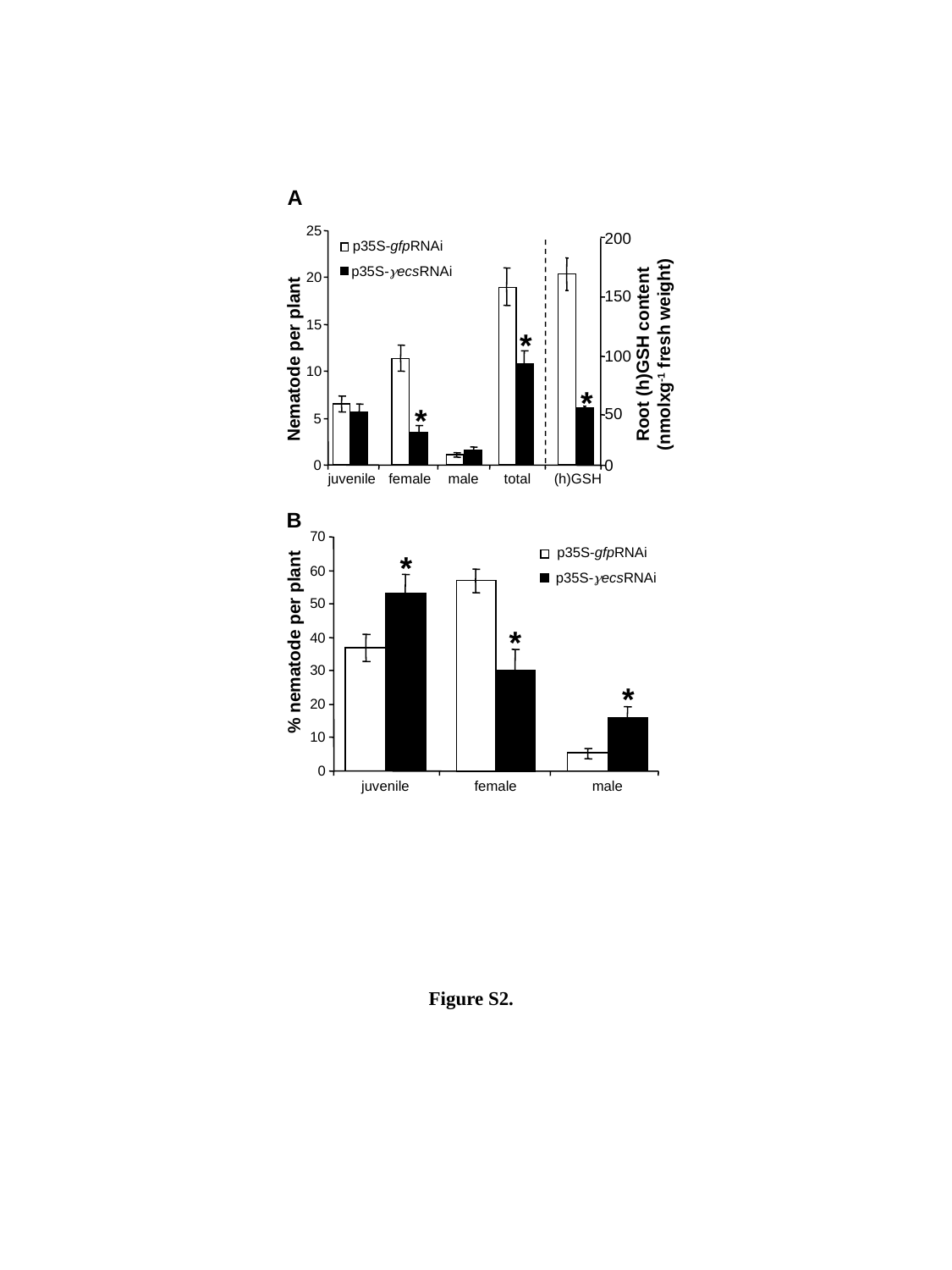

A
25
200
p35S-gfpRNAi
p35S-γecsRNAi
20
150
15
*
Root (h)GSH content (nmolxg-1 fresh weight)
Nematode per plant
100
10
*
*
50
5
0
0
(h)GSH
juvenile
female
male
total
B
70
*
p35S-gfpRNAi
60
p35S-γecsRNAi
50
*
% nematode per plant
40
30
*
20
10
0
juvenile
male
female
Figure S2.
