# Supplementary material for: (Homo)glutathione Deficiency Impairs Root-knot Nematode Development in Medicago truncatula
Source: PLoS Pathog. 2012 Jan 5;8(1):e1002471. doi: 10.1371/journal.ppat.1002471 (PMC3252378; doi:10.1371/journal.ppat.1002471)
Supplement: Table S1 — Mean concentration of individual metabolites in p35S- gfp RNAi and p35S- γecs RNAi galls. Mean of 3 replicates ±standard error. * indicates statistical difference (P<0.05) between p35S-gfpRNAi and p35S- γecsRNAi galls. (DOC) [file ppat.1002471.s005.doc]

**Table S1. Mean concentration of individual metabolites in control RNAi and RNAi-γECS galls.**

|  | Quantification µmol/g FW | |
| --- | --- | --- |
|  | p35S-*gfp*RNAi | p35S-*gecs*RNAi |
| (h)GSH | 0.21 ± 0.02 | 0.06 ± 0.01 ^*^ |
| glucose | 0.29 ± 0,05 | 0 .13 ± 0.05 ^*^ |
| malate | 2.02 ± 0.18 | 0.76 ± 0.24 ^*^ |
| Starch | 1.93 ± 0.15 | 0.69 ± 0.18 ^*^ |

Mean of 3 replicates ±standard error. ^*^ indicates statistical difference (P<0.05) between control RNAi and RNAi γECS galls.
