# Supplementary material for: (Homo)glutathione Deficiency Impairs Root-knot Nematode Development in Medicago truncatula
Source: PLoS Pathog. 2012 Jan 5;8(1):e1002471. doi: 10.1371/journal.ppat.1002471 (PMC3252378; doi:10.1371/journal.ppat.1002471)
Supplement: Supplementary file 4 [file ppat.1002471.s006.doc]

Table S2. List of primers used for the construction of the genetic vectors and to quantify the gene expression levels.

Gene annotation	TC TIGR or GenBank accession number	Primer forward	Primer reverse
Gamma Glutamylcysteine synthetase	AF041340	CTAGAGCTGGCAAAGGATGG	CTCTCAGCTGGAGTGACACCT
Glutathione synthetase	AF075700	CAGTGCGAGACACCCTCATA	GCAACCACCATTACGCATC
Homoglutathione synthetase	AF194421	CCTCACATGATGAAACCAACTC	CAAGCAGGTCGCTGTCTCTT
Patatin-like	TC100534	CGGCTTTAGTTGCCATGAAT	TTGCTGTCCCAGTGCCTATT
Pathogenesis related protein 1	TC143937	TTCGGGTTGGATGTGCTAAG	GGTTGAAGCTCAATGGCACT
Expansin	TC150712	TTGGTGTAACCCTCCTCGTC	CGGTAAGCAACAGGGACAAT
Histone H3	TC147879	ACCGTTGCTCTTCGTGAGAT	ACAAGACGCTGGAATGGAAG
Ribulose-Phosphate 3-epimerase	TC161068	TGGTTCTGCTGTGTTTGGAG	ATTGGCCTGCTGACCATAAA
Cytosolic malate dehydrogenase	TC142639	TCCCTGTTACCTGTGCCAAT	CAGCTCTTCGGCTGTCAAGT
Mitochondrial Malate dehydrogenase	TC137586	CATATTTGCTGATGCTTGCC	TCTTCCCAATCCTCACCTTG
Malate synthase	TC154176	TGGGCAGCACACCCTGGTCTAA	TTGCAGCGTCTTCGCGTTTCA
Sucrose synthase	Hohnjec et al. 2003	ATTCAACTTGTAGGTCTCGAT	GTTGAGAATGAAGAGCACATA
Cell-wall invertase	TC159247	CATCACAGCTAGAGTTTATCCT	TTTGCTTTCTTCATGCTCC
ADP-glucose pyrophosphorylase	TC142140	AGGACACTGTAATGATGGG	CTGACTTTGCTGTTACTTCC
Starch synthase	TC166520	AACTGTACCTATTGTTGCCTC	ACATTGAAGGAACCCATCTG
Alpha-1 4 glucan phosphorylase	TC158103	CAGATTGGAACTTTAGATGGAG	TTTCTTTCCTCAGCCCAG
Asparagine synthase	TC143838	AATGATCTTTGAGAGGTTCTTCCC	AGAAGCATCCCACTCAACAG
Asparaginase	TC146641	GGTTGTGCTTCTGAGGATGG	TCACTGGCAACTAAGTCAGTAACA
Phosphoenol pyruvate carboxylase	TC141881	ACTTGAAGCAAAGACTCAGACTC	CGCACCTCCACCTTGTAGTT
Phosphoenol pyruvate carboxykinase	TC115508	AGGGTGATGACAACCTCACAG	AGTCCCACACACAACACAACA
Pyruvate kinase	TC113792	CTTCACTAGATCGGGTTTCA	CACCTTGATACAAAGCCAAC
MTC27	TC106535	TTACTCAGAGTTGAGACTATG	TCAAACCTGACAACTGACATTC
40S Ribosomal Protein	TC100533	TTGTCCAAGTTTGATGCTGCAC	TCCTACCAACTTCAAAACACCG
γ-ECS-RNAi	AF041340	AAAAAGCAGGCTTCCCATATTCGG AAAACCTCCA	AGAAAGCTGGGTGTCCAATTCCCATC TCCTCAG
